# Small ubiquitin-related modifier 2/3 interacts with p65 and stabilizes it in the cytoplasm in HBV-associated hepatocellular carcinoma

**DOI:** 10.1186/s12885-015-1665-3

**Published:** 2015-10-12

**Authors:** Jun Liu, Manqi Sha, Qianfeng Wang, Yong Ma, Xiaoping Geng, Yufeng Gao, Lijie Feng, Yujun Shen, Yuxian Shen

**Affiliations:** 1 School of Pharmacy, Anhui Medical University, 81 Meishan Road, Hefei, 230032 PR China; 2 Biopharmaceutical Research Institute, Anhui Medical University, 81 Meishan Road, Hefei, Anhui PR China; 3 School of Basic Medical Sciences, Anhui Medical University, 81 Meishan Road, Hefei, 230032 PR China; 4 The Chinese People’s Liberation Army 123 Hospital, 1052 Yanshan Road, Bengbu, 233015 PR China; 5 The First Affiliated Hospital, Anhui Medical University, 218 Jixi Road, Hefei, 230022 PR China; 6 The Second Affiliated Hospital, Anhui Medical University, 678 Furong Road, Hefei, 230601 PR China

**Keywords:** p65, SUMO, SUMOylation, NF-κB, Hepatocellular carcinoma

## Abstract

**Background:**

SUMOylation, an important post-translational modification, associates with the development of hepatocellular carcinoma (HCC). p65, one of the most important subunits of NF-κB, is a key regulator in the development of HCC and has been reported to be SUMOylated by exogenous small ubiquitin-related modifier 3 (SUMO3) in HEK 293T cells. However, the relationship between p65 and SUMO2/3 in HCC remains unknown. This study was to investigate the interaction between p65 and SUMO2/3 and explore the potential roles involved in HCC.

**Methods:**

The expressions of p65 and SUMO2/3 in the liver tissues were detected by using immunohistochemistry. We performed double-labeled immunofluorescence and co-immunoprecipitation assay to verify the interaction between p65 and SUMO2/3. The extraction of nuclear and cytoplasmic proteins was performed, and the subcellular localization of p65 was detected. The proliferation and migration of hepatoma cells were observed using MTT, colony formation, and transwell assays.

**Results:**

We found a strong SUMO2/3-positive immunoreactivity in the cytoplasm in the non-tumor tissues of HCC. However, SUMO2/3 level was down regulated in the tumor tissues as compared with the adjacent non-tumor tissues. In accordance with this finding, p65 was up regulated in the adjacent non-tumor tissues and almost localized in the cytoplasm. There was a close correlation between SUMO2/3 and p65 expressions in the liver tissues (R = 0.800, *p* = 0.006). The interaction between p65 and SUMO2/3 was verified by co-immunoprecipitation and double-labeled immunofluorescent assays. TNF-α (10 ng/ml) treatment for 30 min not only up regulated the cytoplasmic conjugated SUMO2/3, but also enhanced SUMO2/3-p65 interaction. Furthermore, we found that SUMO2/3 up regulated the cytoplasmic p65 protein level in a dose-dependent manner, but not affected its mRNA level. The increase of p65 protein by SUMO2/3 was abolished by MG132 treatment, a reversible inhibitor of proteasome. Meanwhile, TNF-α-induced increase of SUMO2/3-conjugated p65 was along with the reduction of the ubiquitin-conjugated p65. The further study showed that SUMO2/3 over-expression decreased the proliferative ability of hepatoma cells, but did not affect the migration.

**Conclusion:**

SUMO2/3-p65 interaction may be a novel mechanism involved in the transformation from chronic hepatitis B to HCC via stabilizing cytoplasmic p65, which might shed light on understanding the tumorigenesis and development.

**Electronic supplementary material:**

The online version of this article (doi:10.1186/s12885-015-1665-3) contains supplementary material, which is available to authorized users.

## Background

Small ubiquitin-related modifier (SUMO) proteins are a family of small proteins which resemble the three-dimensional structure of ubiquitin [1]. There are four SUMO paralogs in human genome, and they can be found in liver tissues except SUMO4 [2]. Human SUMO2 and SUMO3 share ~97 % sequence and presently cannot be distinguished by antibodies [3], collectively named as SUMO2/3 [4]. SUMO proteins participate in an important post-translational modification called SUMOylation, which promotes SUMO proteins binding to target proteins via an ATP-consuming enzyme cascade, including SUMO-activating enzyme (SAE1/SAE2), Ubiquitin-conjugating enzyme 9 (Ubc9), and several E3 ligases [5]. SUMOylation is involved in regulation of diverse cellular processes, such as transcriptional regulation, subcellular localization, target proteins stability, and maintenance of genome integrity [6]. Meanwhile, a number of studies have shown that SUMOylation also plays an important role in human pathogenesis, especially inflammation-related cancer, such as hepatocellular carcinoma (HCC) [7, 8]. Moreover, SENP2, one of the most important de-SUMOylation proteases, plays a key role in the control of HCC cell growth and functions as a tumor suppressor [9, 10]. Ubc9, the only E2 conjugating enzyme in the SUMOylation cycle that transfers the activated SUMO proteins to target proteins, is increased in HCC [11].

NF-κB pathway is important for the development of HCC, which acts as a central link in this process [12]. In other words, abnormal activation of the NF-κB pathway is closely related to hepatocarcinogenesis [13–15]. For instance, HBx protein can induce HBV-related HCC by activating NF-κB pathway [13]. Meanwhile, increasing the NF-κB signaling promotes motile and invasive abilities of HCC cells [14]. Furthermore, the NF-κB pathway reduces the sensitivity of HCC cells to radiotherapeutics and chemotherapeutics [15]. On the contrary, inhibition of NF-κB pathway contributes to prevent the motility and invasiveness of human HCC cells and reduces carcinogenesis in vivo [16, 17].

p65, one of the most important subunits of NF-κB, is a critical regulator of NF-κB transcriptional activity and associates with the development of HCC. Nuclear p65, as an active form, can induce a wide array of genes transcription in response to inflammatory stimuli [18]. HBx protein promotes p65 nuclear translocation and leads to liver tumorigenesis [19]. Therefore, the regulation of p65 stability is very important for hepatitis-related HCC. Recently, it has been verified that p65 could be SUMOylated by exogenous SUMO3 in HEK 293T cells and mouse 3T3 fibroblast cells [20]. Moreover, SUMO2/3 knockdown increased TNF-α-induced transcriptional activity of p65 in Hela cells [21]. However, the studies about the relationship between SUMO2/3 and p65 are limited.

In current study, we investigated the expression and subcellular localization of SUMO2/3 and p65 in human liver samples and found a close correlation between them. Then, we verified the interaction between p65 and SUMO2/3, and found TNF-α promoted their interaction. Meanwhile, SUMO2/3-modified p65 SUMOylation was detected in vitro hepatoma cell lines and in vivo human liver tissues as well. The mechanisms by which SUMO2/3 stabilizes cytoplasmic p65 were explored. Finally, we observed the effects of SUMO2/3 on the proliferation and migration of hepatoma cells.

## Methods

### Human liver specimens and ethics statement

The human liver tissues from 5 patients with hepatitis (early stage, without fibrosis) and 8 patients with HCC were used in this study. These specimens were obtained from the First Affiliated Hospital of Anhui Medical University (AMU), the Second Affiliated Hospital of AMU, and the Chinese People’s Liberation Army 123 Hospital. The use of clinical HCC specimens was in accordance with the Declaration of Helsinki and was approved by the Ethics Committee of AMU (No. 20131359).

### Cells and plasmids

HepG2 cells and SMMC7721 cells were purchased from the Type Culture Collection of the Chinese Academy of Sciences, Shanghai, China. GFP-SUMO2 and GFP-SUMO3 constructs were kindly gifted by Prof. Steve Jackson (University of Cambridge, UK) [22]. Myc-Ubc9 plasmid was kindly gifted by Prof. Hiderou Yoshida (University of Hyogo, Japan) [23].

### Reagents and antibodies

MG132, N-Ethylmaleimide (NEM) and anti-tubulin antibody were purchased from Sigma-Aldrich (St. Louis, MO, USA). Antibodies against p65, SUMO2/3, and histone H3 were purchased from Abcam (Cambridge, MA, USA). Rabbit IgG was obtained from cell signaling technology (Bevery, MA, USA). Alexa Fluor 488-conjugated or 568-conjugated IgG, Trizol reagent, Lipofectamine 2000, and Opti-MEM were obtained from Invitrogen (Carlsbad, CA, USA). Horseradish peroxidase (HRP)-conjugated streptavidin was purchased from Zhongshan Biotechnical Company (Beijing, China). TNF-α was purchased from R&D Systems. Complete™ protease inhibitors were obtained from Roche Applied Science. Polymerase chain reaction (PCR) Master Mix was obtained from TaKaRa Biotechnology (Dalian, China). Pierce Protein A agarose was purchased from Thermo (Rockford, IL61001, USA). Nuclear and cytoplasmic protein extraction kit was purchased from Beyotime Institute of Biotechnology (Shanghai, China). SUMO2/3 and NC (negative control) siRNA were synthesized and purified by GenePharma (Shanghai, China).

### Immunohistochemistry and the integral optical density (IOD) measure

The liver samples were paraformaldehyde-fixed, paraffin-embedded, and serially sectioned. Following dimethylbenzene and rehydration with graded ethanol, antigen retrieval was performed using microwave irradiation. Endogenous peroxidase was quenched with 10 % H_2_O_2_ at 37 °C for 10 min. After incubated with goat serum for 30 min to block the non-specific binding, the sections were incubated with the corresponding primary antibodies at 37 °C for 2 h and secondary antibody for 30 min. Immunostaining was performed by application of 3,3′-diaminobenzidine tetrahydrochloride (DAB). The sections were counterstained with hematoxylin, dehydrated in graded ethanol, cleared in dimethylbenzene, and then observed under microscope. We selected one section per patient to observe and switched objective from low magnification to high magnification under bright field illumination. For the quantitative analysis, we used Image-Pro Plus software to measure IOD [24] in six independent high-magnification (40× objective) fields per section, and the average was calculated.

### Dual fluorescence staining

After antigen retrieval, liver sections were blocked with 5 % goat serum. The sections were incubated with primary antibodies which were dissolved in PBS containing 0.5 % Triton X-100 overnight at 4 °C. The sections were then incubated with Alexa Fluor 488-conjugated or 568-conjugated IgG for 1 h at 37 °C. Meanwhile, the nuclei were stained with 4′,6-diamidino-2-phenylindole (DAPI), and the images were collected by fluorescence microscopy.

### Immunocytochemistry

The cells planted on coverslips were fixed with 4 % paraformaldehyde at room temperature for 15 min, permeabilized in 0.1 % Triton X-100 for 15 min, blocked by 5 % BSA at 37 °C for 30 min, incubated with primary antibodies at 37 °C for 2 h, and stained with Alexa Fluor 488-conjugated or 568-conjugated IgG at 37 °C for 1 h. Meanwhile, the nuclei were stained with DAPI, and the images were collected using fluorescence microscopy.

### Proteins extraction and western blot

The proteins in whole cell were extracted using RIPA lysis buffer. Nuclear and cytoplasmic proteins were extracted by using the nuclear and cytoplasmic protein extraction kit according to the manufacturer’s instructions. A total amount of 10–15 μg protein in each sample was separated by 10 %–15 % SDS-PAGE gel and then transferred to PVDF membranes. After blocking with 5 % nonfat milk, the proteins were detected by the corresponding antibodies.

### Co-immunoprecipitation

The liver tissues and cells were lyzed in a lysis buffer containing 50 mM HEPES (pH 7.4), 150 mM NaCl, 2 mM EGTA, 0.1 % Triton X-100, NEM, and Complete™ protease inhibitors. The lysates were centrifuged at 12000 rpm for 20 min. The supernatants were collected, and then mixed with Protein A agarose and various antibodies for 4 h at 4 °C. Protein A agarose was then eluted and the bound proteins were analyzed using western blot.

### RT-PCR

Total RNA was extracted from SMMC7721 cells with Trizol reagent according to the manufacturer’s protocol, and then was reversely transcripted into cDNA with a PrimeScript™ RT Reagent Kit at 37 °C for 30 min. The primer sequences for p65 and β-actin were as follows: p65 forward primer, 5′-AACAACAACCCCTTCCAA-3′, and reverse primer, 5′-TGTCCTCTTTCTGCACCTT-3′ (product size 262 bp); β-actin forward primer, 5′-TCACCAACTGGGACGACAT-3′, and reverse primer, 5′-GCACAGCCTGGATAGCAAC-3′ (product size 192 bp).

### MTT assay

The viability of SMMC7721 cells was quantitatively assessed by MTT assay at 48 h post-transfection with plasmids or siRNA. The cells were incubated in 500 mg/ml MTT solution for 4 h. After solubilization of formazan crystals in DMSO, the optical density of each well was determined by a spectrophotometric reader at 570 nm (Rayto, RT-2100c, USA).

### Colony formation assay

SMMC7721 cells were transfected with pEGFP-C1, GFP-SUMO2, GFP-SUMO3, NC-siRNA, and SUMO2/3-siRNA, respectively. Forty-eight hrs later, 200 cells were planted in a six-well plate. Twelve days later, the cells were stained with crystal violet. For the quantitative analysis, the positive colonies were counted in the whole well. The data were expressed as the means ± SD from three independent experiments.

### Migration assay

SMMC7721 cells were transfected with the plasmids or siRNA. Twenty-four hrs later, 4 × 10^4^ cells in serum-free medium were seeded in the upper compartment of a transwell chamber (Corning, Lowell, MA). After incubation for 24 h, the migrated cells on the lower membrane were stained with 0.1 % crystal violet and 20 % methanol. The positive colonies were counted from four fields of one membrane under microscope (20× objective), and the average was calculated. The data were expressed as the means ± SD from three independent experiments.

### Statistical analysis

Quantitative data were expressed as the means ± SD. Statistical comparisons were performed using a one-way ANOVA followed by the Dunnett’s test. Independent-samples T test was used to compare the means from two groups. Paired-Samples T test was used to compare the means of protein levels in the liver tissues of HCC patients. Pearson’s correlation test was used to assess the correlations of two proteins. *p* < 0.05 was considered statistically significant.

## Results

### Correlation between SUMO2/3 and NK-κB p65 in human liver tissues

**Fig. 1:**
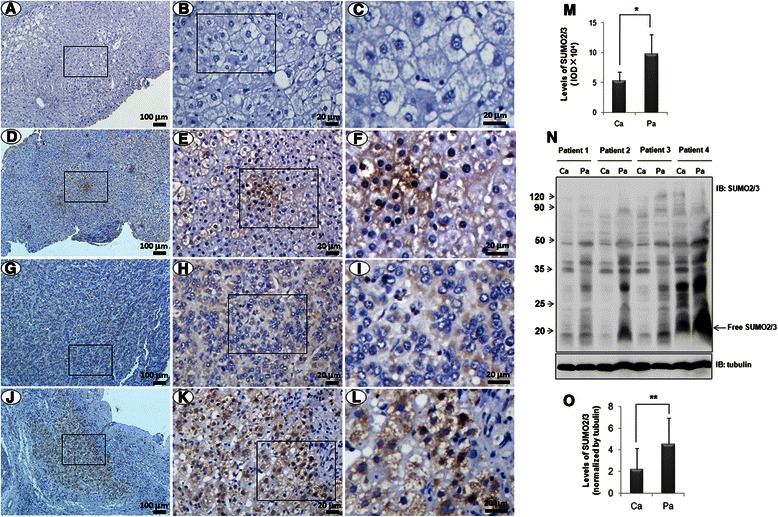
The profile of SUMO2/3 expression in the liver tissues. SUMO2/3 expression was detected using immunohistochemistry in the liver tissues of hepatitis B (**d-f**), HCC (**g-i**), and corresponding adjacent non-tumor tissues (**j-l**). **a-c** The isotype IgG control. **b**, **e**, **h**, and **k** are the magnified images of the rectangles in **a** , **d**, **g**, and **j**, respectively. **c**, **f**, **i**, and **l** are the magnified images of the rectangles in **b**, **e**, **h**, and **k**, respectively. The scale bars were shown as indicated. **m** The integral optical density (IOD) in panel **g**-**l**. The data were expressed as the means ± SD of the 5 individuals (*n =* 5). *p < 0.05, compared with Pa. Ca: tumor; Pa: para-tumor. **n** The levels of SUMO2/3 were detected by western blot. **o** The quantitative data in panel **n**. The data were expressed as the means ± SD of the 4 individuals (*n =* 4). **p < 0.01, compared with Pa. Ca: tumor; Pa: para-tumor

**Fig. 2:**
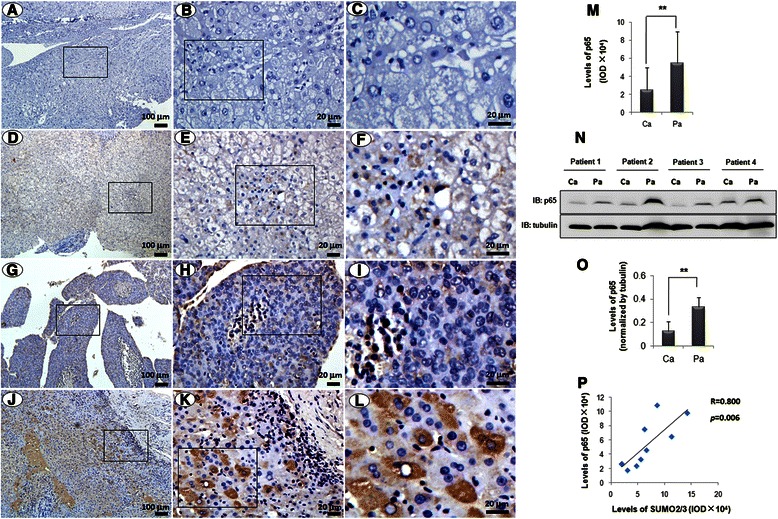
The profile of p65 expression in the liver tissues. p65 expression was detected using immunohistochemistry in the liver tissues of hepatitis B (**d-f**), HCC (**g-i**), and corresponding adjacent non-tumor tissues (**j-l**). **a-c** The isotype IgG control. **b**, **e**, **h**, and **k** are the magnified images of the rectangles in **a**, **d**, **g**, and **j**, respectively. **c**, **f**, **i**, and **l** are the magnified images of the rectangles in **b**, **e**, **h**, and **k**, respectively. The scale bars were shown as indicated. **m** The integral optical density (IOD) in panel **g-l**. The data were expressed as the means ± SD of the 8 individuals (*n =* 8). *p < 0.05, compared with Pa. Ca: tumor; Pa: para-tumor. **n** The levels of p65 were detected by western blot. **o** The quantitative data in panel **n**. The data were expressed as the means ± SD of the 4 individuals (*n =* 4). **p < 0.01, compared with Pa. Ca: tumor; Pa: para-tumor. **p** Pearson’s correlation test was used to analyse the relationship between the IODs of SUMO2/3 and p65 in the human liver tissues. *p* = 0.006, *n =* 9, R = 0.800

Previous study showed that p65 and SUMO proteins were key regulators in the development of HCC [25, 26]. Therefore, we want to observe the characteristics of SUMO2/3 and p65 proteins in human liver tissues. Immunohistochemistry assay showed that SUMO2/3 was remarkably increased in the adjacent non-tumor tissues, compared with that in the tumor tissues, and SUMO2/3 was mainly localized in the cytoplasm (Fig. 1g–l, m). Western blotting assay also showed SUMO2/3 was significantly increased in the para-tumor (Fig. 1n–o). In the early stage of hepatitis B, only a few cells were SUMO2/3-positive, and SUMO2/3 mainly localized in the nuclei (Fig. 1d–f). Similarly, a few p65-positive cells appeared in the liver tissues of hepatitis B at early stage, where p65 was found to localize in nucleus and cytoplasm of the hepatocytes (Fig. 2d–f). However, p65 was largely expressed in the non-tumor tissues and mainly localized in the cytoplasm (Fig. 2j–l, m), although it was also detectable in the tumor tissues (Fig. 2g–i). Western blotting assay also showed p65 was significantly increased in the para-tumor (Fig. 2n–o). These results suggest that the expression and distribution of p65 are diverse in hepatitis and HCC.

**Fig. 3:**
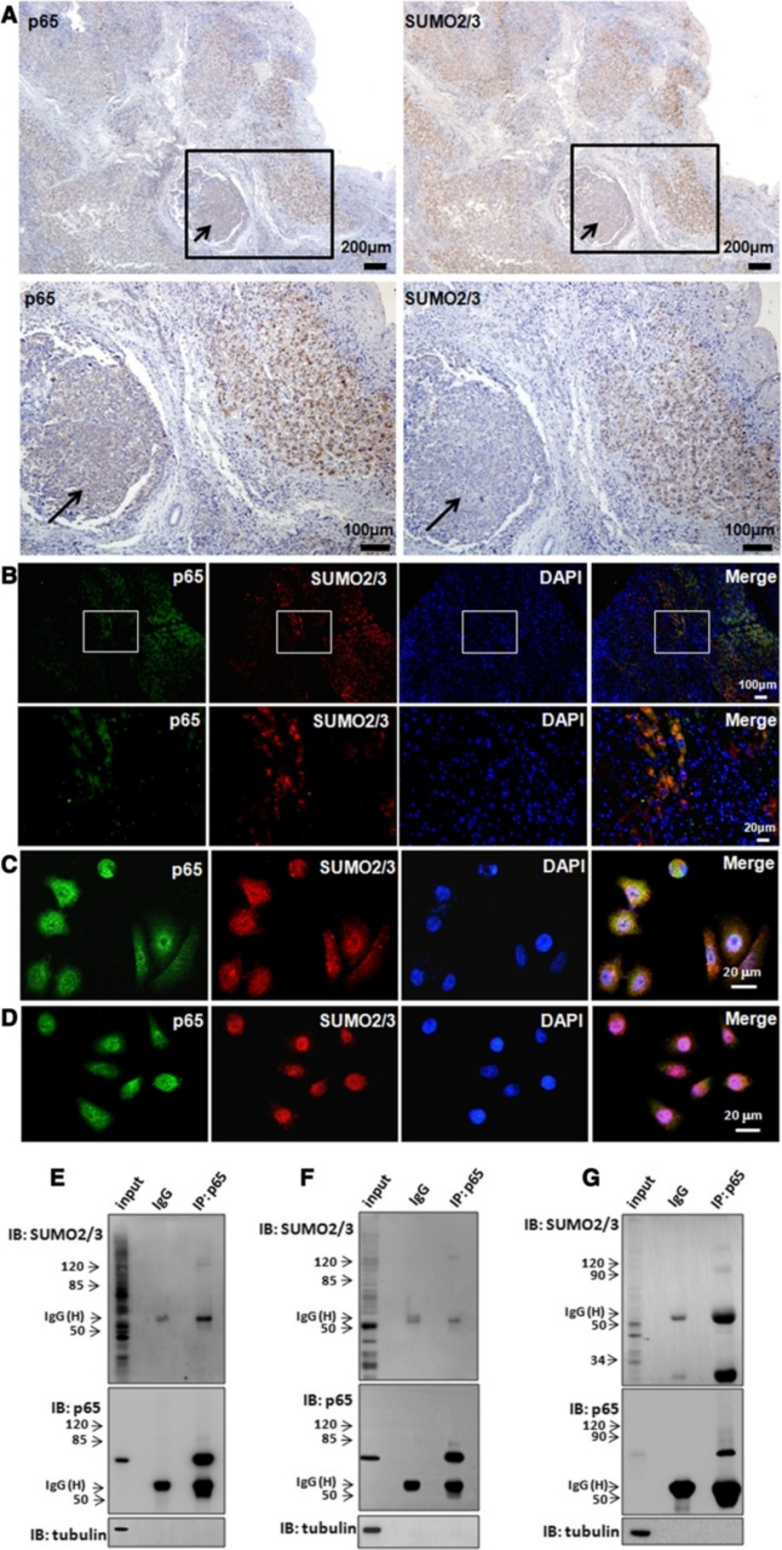
SUMO2/3 interacts with p65 in liver tissues and hepatoma cell lines. **a** The similar profiles of p65 and SUMO2/3 in the liver tissues. p65 and SUMO2/3 were detected using immunohistochemistry in the serial sections of the liver tissues. The images of the rectangles in the upper panels are magnified in the lower panel. The scale bars were shown as indicated. The arrows indicate the tumor tissues. **b-d** Co-localization of SUMO2/3 and p65. Double-labeled immunofluorescent staining was performed using the antibodies against p65 (green) and SUMO2/3 (red) in the liver tissues (**b**), HepG2 cells (**c**) and SMMC7721 cells (**d**). The nuclei were stained with DAPI (blue). In panel **b**, the images of the rectangles in the upper panels are magnified in the lower panels. The scale bars were shown as indicated. **e**-**f** Interaction of p65 and SUMO2/3 in the hepatoma cells. SMMC7721 cells were co-transfected with myc-Ubc9 and GFP-SUMO2 (**e**) or GFP-SUMO3 (**f**). At 24 h after transfection, the cells were lyzed and incubated with Protein A agarose containing anti-p65 antibody. **g** Interaction of p65 and SUMO2/3 in the liver tissues. The non-tumor tissues collected from HCC patients were lyzed and co-immunoprecipitation assay was performed using anti-p65 antibody. The isotype IgG was used as a negative control. The bound proteins were blotted by using the antibodies as indicated. 2 % total lysate was loaded as input

The similar cytoplasmic characteristics of SUMO2/3 and p65 in liver tissues imply a correlation between them. To confirm it, we observed the expressions of SUMO2/3 and p65 in the serial sections of HCC. Fortunately, we could see tumor and non-tumor tissues in the same field under microscope. The results showed that the levels of SUMO2/3 and p65 were decreased in the tumor tissues (Fig. 3a, indicated by arrows), compared with that in the non-tumor tissues. We also found there was a close correlation between SUMO2/3 and p65 expressions in the liver tissues (Fig. 2p, R = 0.800, *p* = 0.006).

### SUMO2/3 interacts with p65 in the liver tissue and hepatoma cell lines

**Fig. 4:**
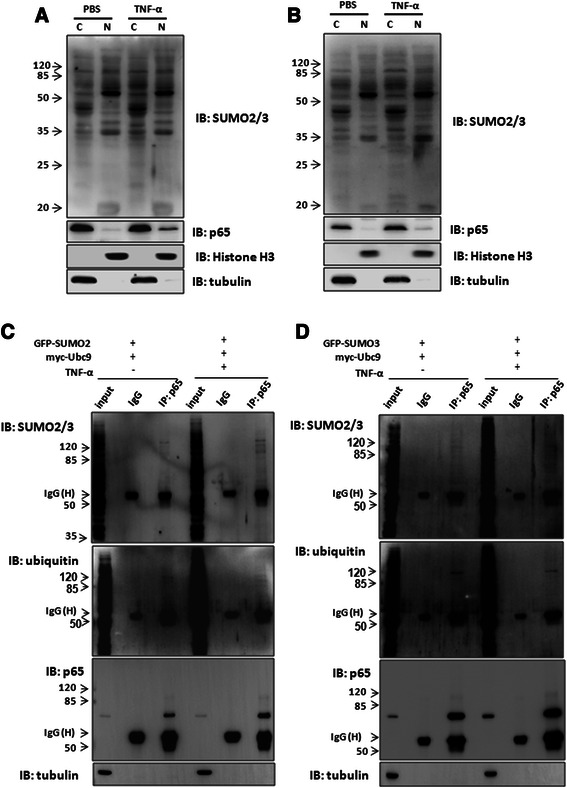
TNF-α up-regulates SUMO2/3 and promotes p65 SUMOylation. **a**-**b** SMMC7721 cells were treated with TNF-α (10 ng/ml) for 30 min (**a**) and 8 h (**b**), respectively. The nuclear (N) and cytoplasmic (C) fragments were extracted by using the protein extraction kit according to the manufacturer’s instruction. The proteins were blotted with the antibodies as indicated. Tubulin and histone H3 were used as the markers of cytoplasm and nucleus, respectively. **c-d** SMMC7721 cells were co-transfected with myc-Ubc9 and GFP-SUMO2 (**c**) or GFP-SUMO3 (**d**). At 24 h after transfection, the cells were treated with TNF-α (10 ng/ml) for 30 min. Then the cells were lyzed and incubated with anti-p65 antibody. The isotype IgG was used as a negative control. The bound proteins were blotted by using the antibodies as indicated. 2 % total lysate was loaded as input

Double-labeled immunofluorescent assay showed that SUMO2/3 was co-localized with p65 in the cytoplasm in the non-tumor tissues (Fig. 3b). Additionally, we found SUMO2/3 was co-localized with p65 in HepG2 cells (Fig. 3c) and SMMC7721 cells (Fig. 3d). Co-immunoprecipitation assay showed that there was a weak interaction between p65 and SUMO2/3 in the SMMC7721 cells over-expressing SUMO2 or SUMO3 (Fig. 3e–f). The interaction of p65 and SUMO2/3 was found in the liver tissues (Fig. 3g). Additionally, the interaction between p65 and SUMO2/3 was enhanced by TNF-α treatment for 30 min (Fig. 4c–d, lane 6 *vs* lane 3), suggesting that inflammatory response may strengthen the interaction of SUMO2/3 and p65.

### TNF-α up-regulates SUMO2/3 protein level

Because there was a chronic inflammatory response in the non-tumor tissue, we wondered whether the up-regulation of SUMO2/3 is associated with inflammatory response or not. To test this issue, we treated SMMC7721 cells with TNF-α (10 ng/ml) for 30 min or 8 h. The results showed that the nuclear p65 was obviously up-regulated at 30 min, other than at 8 h after TNF-α treatment. TNF-α treatment for 30 min caused the conjugated SUMO2/3 increase in the cytoplasm (Fig. 4a, lane 3), whereas TNF-α treatment for 8 h caused a slight increase in cytoplasmic and nuclear SUMO2/3 (Fig. 4b). These results suggest that the induction of SUMO2/3 and p65 depends on the time of inflammatory stimulation.

### TNF-α enhances SUMO2/3-modified p65 SUMOylation

It has been newly reported that p65 was SUMOylated by exogenous SUMO3 in HEK 293T cells and mouse 3T3 fibroblast cells [20]. We wondered whether inflammatory response enhances SUMO2/3-modified p65 SUMOylation or not. To test this issue, we performed a co-immunoprecipitation assay using anti-p65 antibody, and detected the SUMO2/3-conjugated p65 using anti-SUMO2/3 antibody. The result showed that there was a weak interaction between p65 and SUMO2/3 (Fig. 4c–d, lane 3). However, TNF-α (10 ng/ml, 30 min) treatment increased the conjugated SUMO2/3 (Fig. 4c–d, lane 6). Therefore, TNF-α can strengthen SUMO2/3-p65 interaction and the SUMO2/3-modified p65 SUMOylation.

### SUMO2/3 regulates the stability of cytoplasmic p65

**Fig. 5:**
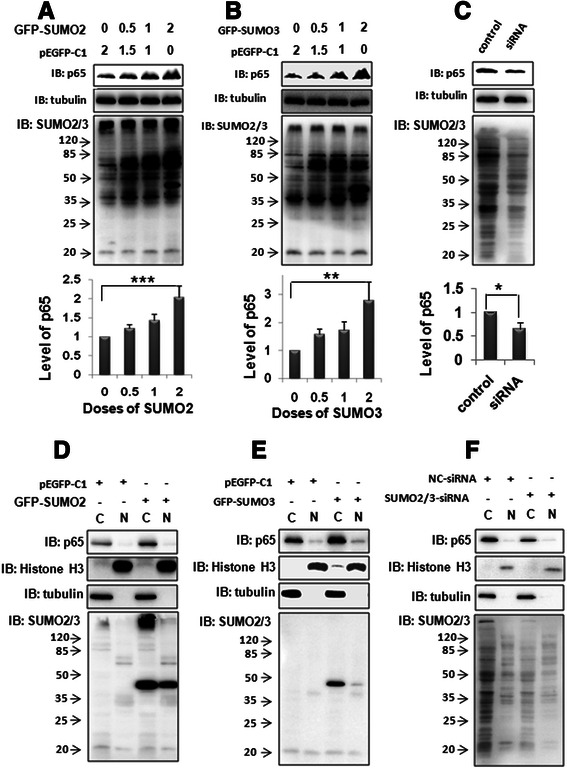
SUMO2/3 stabilizes the cytoplasmic p65. **a**-**b** Dose-dependent effects of SUMO2/3 on p65 level. SMMC7721 cells were transfected with GFP-SUMO2 (**a**) or GFP-SUMO3 (**b**). The blank vector (pEGFP-C1) was transfected to balance the total DNA. At 24 h after transfection, the cells were collected and processed for immunoblotting. The data were expressed as the means ± SD of at least three independent experiments. **p < 0.01, ***p < 0.001, compared with pEGFP-1 vector. **c** SUMO2/3 knockdown decreased p65 level. SMMC7721 cells were transfected with SUMO2/3-siRNA (siRNA) or negative control siRNA (control). Western blotting assay was performed 48 h after transfection. The data were expressed as the means ± SD of at least three independent experiments. *p < 0.05, compared with the control. **d-e** SUMO2/3 increased the cytoplasmic p65. SMMC7721 cells were transfected with GFP-SUMO2 (**d**) or GFP-SUMO3 (**e**). The blank vector (pEGFP-C1) was transfected to balance the total DNA. Nuclear and cytoplasmic proteins were extracted by using the protein extraction kit according to the manufacturer’s instructions. Tubulin and histone H3 were used as the markers of cytoplasm and nucleus, respectively. **f** SUMO2/3 knockdown decreased the cytoplasmic p65. SMMC7721 cells were transfected with SUMO2/3-siRNA. NC (negative control) siRNA was used as a control. Nuclear and cytoplasmic proteins were extracted 48 h after transfection and processed for Western blotting

We have observed that both SUMO2/3 and p65 levels were increased in the non-tumor of liver, where they co-localized in the cytoplasm. Therefore, we want to know whether SUMO2 or SUMO3 affects p65 level. To answer this question, we transfected SMMC7721 cells with the different doses of GFP-SUMO2 or GFP-SUMO3, and performed a Western blotting assay to detect p65 level. The results showed that SUMO2 or SUMO3 over-expression increased p65 level in a dose-dependent manner (Fig. 5a–b). On the contrary, knockdown of endogenous SUMO2/3 reduced p65 protein level (Fig. 5c). These results suggest that SUMO2/3 stabilizes the total level of p65.

We further isolated the nuclear and cytoplasmic fragments and found that either SUMO2 or SUMO3 over-expression increased the cytoplasmic p65 (Fig. 5d–e, lane 3), but not the nuclear p65 (Fig. 5d–e, lane 4). Consistently, knockdown of endogenous SUMO2/3 reduced the cytoplasmic p65 (Fig. 5f, lane 3). These results suggest that SUMO2/3 stabilizes the cytoplasmic p65 level.

### Ubiquitin-proteasome system is involved in SUMO2/3-mediated stability of the cytoplasmic p65

**Fig. 6:**
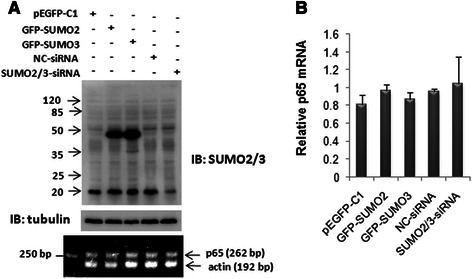
Effect of SUMO2/3 on p65 mRNA expression. SMMC7721 cells were transfected with the plasmids or siRNA as indicated. The expressions of SUMO2/3 proteins and p65 mRNA were detected using Western blotting and RT-PCR, respectively (**a**). The relative mRNA was quantitative analyzed in (**b**). The data were expressed as the means ± SD of at least three independent experiments

To figure out the reasons that cause p65 increase in the cytoplasm, we investigate the effect of SUMO2/3 on p65 mRNA level. As showed in Fig. 6, neither SUMO2/3 over-expression nor knockdown affects p65 mRNA level.

**Fig. 7:**
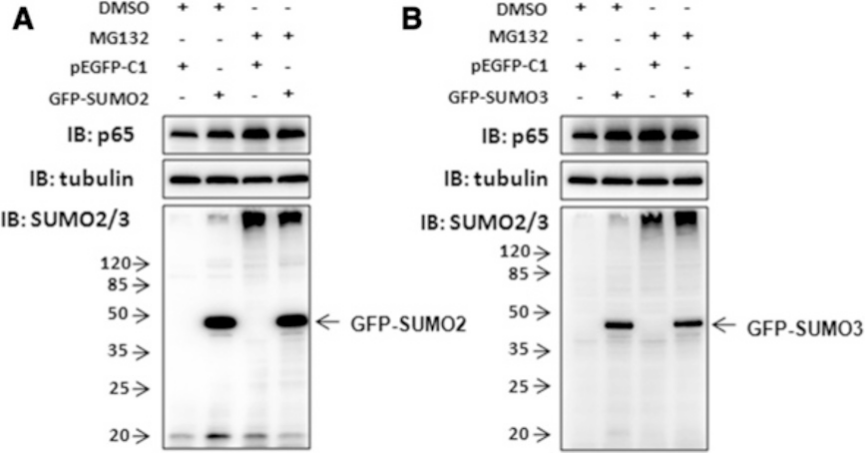
Proteasome inhibitor MG132 abolished SUMO2/3-mediated p65 stability. SMMC7721 cells were transfected with GFP-SUMO2 (**a**) and GFP-SUMO3 (**b**). At 24 h after transfection, the cells were treated with MG132 (20 μM) for 6 h. Then the lysate was subjected to Western blotting analysis

Previous study showed that SUMOylation regulates the stability of target proteins via antagonizing ubiquitination [27, 28]. To explore the mechanism by which SUMO2/3 stabilizes the cytoplasmic p65 level, SMMC7721 cells were transfected with GFP-SUMO2 or GFP-SUMO3. The cells were then treated with a reversible proteasome inhibitor MG132 (20 μM) for 6 h at 24 h after transfection. The result showed that MG132 abolished SUMO2/3-mediated p65 stability (Fig. 7a–b). In Fig. 4, we also observed that the ubiquitin-conjugated p65 were reduced along with the increase of the SUMO2/3-conjugated p65 induced by TNF-α (Fig. 4c–d, lane 6 vs lane 3). These findings suggest that SUMO2/3-mediated stability of cytoplasmic p65 may be related to suppressing p65 degradation via ubiquitin-proteasome system.

### Effects of SUMO2/3 on the proliferation and migration of hepatoma cells

**Fig. 8:**
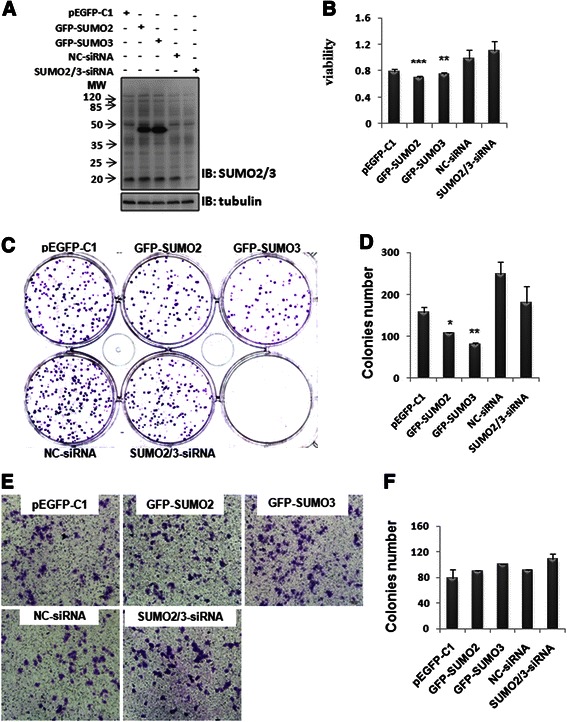
Effects of SUMO2/3 on the proliferation and migration of hepatoma cells. SMMC7721 cells were transfected with SUMO2/3 plasmid or SUMO2/3-siRNA. At 48 h after transfection, Western blot was used to detect SUMO2/3 expression (**a**). The proliferation was determined using MTT assay (**b**) and colony formation assay (**c**-**d**). The migration was detected using transwell assay (**e**-**f**). The data were expressed as the means ± SD of at least three independent experiments. *p < 0.05, **p < 0.01, ***p < 0.001, compared with pEGFP-C1 vector control

NF-κB p65 is an important transcription factor and plays a crucial role in the development of HCC. Our results also showed that SUMO2/3 interacted with p65, and subsequently stabilized p65 in the cytoplasm. It was reported recently that SUMO2/3 knockdown increased TNF-α-induced transcriptional activity of p65 in Hela cells using a luciferase assay [21]. Therefore, we want to know whether SUMO2/3 affects the proliferation and migration of hepatoma cells or not. The results showed that SUMO2 or SUMO3 over-expression significantly decreased the proliferation of SMMC7721 cells (Fig. 8b–d). However, SUMO2/3 knockdown had no effect on the proliferation (Fig. 8b–d). Meanwhile, SUMO2/3 had little effect on the migration of SMMC7721 cells (Fig. 8e–f). These results suggest that the over-expressed SUMO2 or SUMO3 inhibits hepatoma cell proliferation, but does not affect hepatoma cell migration.

## Discussion

In this study, we reported a novel observation that SUMO2/3 interacts with p65 and stabilizes p65 in the cytoplasm. Specifically, SUMO2/3-modified p65 SUMOylation was enhanced by TNF-α. Meanwhile, SUMO2/3 stabilized the cytoplasmic p65 in a dose-dependent manner, which was abolished by the proteasome inhibitor MG132. Further study found that the cytoplasmic stability of p65 is related to the SUMO2/3-modified p65 SUMOylation, the later antagonized the degradation of ubiquitin-conjugated p65. Additionally, SUMO2/3 over-expression inhibited the proliferation of hepatoma cells.

NF-κB is composed of a heterodimer or homodimer of various members (p50, p52, cRel, p65, and RelB), and it is responsible for the activation of many genes required for inflammation and immune response [29, 30]. Previous studies indicated that NF-κB pathway is activated in the liver tissues with HBV infection [19, 31]. HBx interacts with IκBα directly and forms HBx-IκBα complex to promote the nuclear translocation of NF-κB [19, 32]. On the other hand, the activated NF-κB signaling regulates the stability of HBx protein [33]. HBx-NF-κB forms a positive feedback circuit, which might account for the HBV-related carcinogenesis [33]. Therefore, NF-κB pathway is closely associated with chronic hepatitis and HCC [31, 34]. p65, an important subunit of NF-κB transcription complex, has been considered as the central mediator of the inflammatory and tumorous process [35–37]. Aberrant p65 signaling in HCC has been reported. For example, p65 expression in liver tumor tissues is higher than that in the non-tumor tissues [38, 39]. However, our results showed that the cytoplasmic p65 in the tumor tissues was lower than that in the adjacent non-tumor tissues. The difference between our results and others may come from the variety of liver samples. The tissues in the location where is far away from tumors are totally different from that where is very close to the tumors. The structure of tissue and the morphology of hepatocytes may be diverse in the different regions even if the samples were collected from the same patient. The pathological changes may vary along with the duration and severity of hepatitis. Therefore, the pathogenesis of chronic hepatitis B-derived HCC is complicated and various.

In present study, we observed that p65 was largely expressed in the non-tumor tissues, where it mainly localized in the cytoplasm of hepatocytes. Because the liver samples were collected from the HBV-related HCC, we speculate that the surviving hepatocytes might suffer from long-time inflammation, compensatory liver repair, and regeneration or proliferation. Therefore, we assume that the increase of cytoplasmic p65 in the para-carcinoma tissues may be associated with chronic inflammation. It was reported that strong staining of p65 protein was detected in the liver tissue from patients with HBV infection [40], which was consistent with our findings. As compared with the adjacent para-carcinoma tissues, the higher expression of nuclear p65 (an activated form of NF-κB) in the tumor tissues has been reported [41]. To exclude the possibility that the difference was caused by the detection antibody, we determined the subcellular localization of p65 in hepatoma cell lines using immunohistochemical assay. The results showed that p65 was translocated into the nuclei after treatment with TNF-α [see Additional file 1]. This result suggests that the anti-p65 antibody was specific and worked well.

Our results showed that the immunoreactivity of SUMO2/3 was stronger in the non-tumor tissues than that in the tumor tissues, although SUMO2/3 was also up-regulated in the tumor as compared with that in the liver tissues of hepatitis B. Meanwhile, we found SUMO2/3 was predominantly localized in cytoplasm both in the tumor tissues and in the non-tumor tissues. The previous study showed that SUMO2 was mainly distributed in nucleus, while SUMO3 in cytoplasm in Hela cells that were transfected with FLAG-SUMO2 or -SUMO3 [42]. This result was similar to ours. However, we did not distinguish endogenous SUMO2 and SUMO3 in the liver tissues, because we cannot find the commercial antibodies that are specific for SUMO2 and SUMO3. The similar difficulties were reported recently by Barysh et al. [3, 43].

Chronic inflammation in liver is strongly linked to the development of fibrosis, cirrhosis, and HCC [31]. Therefore, in the non-tumor tissue around HCC, the surviving hepatocytes usually suffer from a chronic inflammation. Because all the liver tissues used in present study were collected from the patients with chronic hepatitis B, we assume that SUMO2/3 up-regulation may be caused by inflammatory stimuli. To test this hypothesis, we firstly treated the hepatoma cells in vitro with inflammatory factor TNF-α. We noticed that TNF-α regulated SUMO2/3 expression dependently on the treatment time. Short time treatment with TNF-α increased the cytoplasmic level of the covalent SUMO2/3, whereas long time treatment slightly elevated SUMO2/3 in the cytoplasm and nucleus as well. The previous study showed that SUMO2 was mainly distributed in the nucleus, while SUMO3 in the cytoplasm [42]. We propose that this result is consistent with ours although we did not identify the endogenous SUMO2 and SUMO3 with the specific antibodies (we cannot find the antibodies) [3]. Meanwhile, Kim EM showed that SUMO2 over-expression inhibited p65 translocation into the nucleus [44]. We also found TNF-α enhanced SUMO2/3-p65 interaction. Therefore, we speculate that long time TNF-α treatment might limit p65 nuclear translocation through regulating SUMO2 expression.

In the liver samples, we observed that the immunoreactivity of SUMO2/3 or p65 was higher in the para-carcinoma than that in the carcinoma. Interestingly, the distribution of SUMO2/3-positive cells was closely correlated with that of p65-positive cells. More importantly, both SUMO2/3 and p65 were remarkably up-regulated in the cytoplasm compartment. To explore the association between SUMO2/3 and p65, we over-expressed SUMO2 and SUMO3 in hepatoma cells, respectively. We found that either SUMO2 or SUMO3 increased the total level of p65 in a dose-dependent manner. Consistently, knockdown of endogenous SUMO2/3 reduced the total level of p65. Furthermore, the nuclear and cytoplasmic extraction showed that over-expressions of SUMO2 and SUMO3 up regulated the cytoplasmic p65, and knockdown of endogenous SUMO2/3 down regulated the cytoplasmic p65. Consistently, Kim EM found that retroviral vector containing SUMO2 inhibited the nuclear translocation of p65 [44]. These results demonstrate that SUMO2/3 stabilizes p65 in cytoplasm. However, our results indicated that neither SUMO2/SUMO3 over-expression nor SUMO2/3 knockdown affected p65 mRNA level. Therefore, we proposed that SUMO2/3 stabilizes p65 in protein level. Consistently, MG132, a reversible proteasome inhibitor, abolished the effect of SUMO2/3 on p65 stability. We also found that ubiquitin-conjugated p65 was reduced along with the increase of SUMO2/3-conjugated p65 under TNF-α treatment, suggesting SUMO2/3-modified p65 SUMOylation may inhibit ubiquitin-mediated p65 degradation.

It was known that SUMOylation and ubiquitination exhibit similar biological processes of post-translational modification [1, 45]. Therefore, SUMO proteins compete with ubiquitin for the same lysine and inhibit proteasome-mediated degradation of target proteins [27, 28]. Some studies have shown that the proteasome-mediated p65 degradation often occurs in the nucleus [46, 47]. More research results showed that the E3 ubiquitin ligase PPARγ and ING4 not only induce nuclear p65 degradation but also enhance cytoplasmic p65 degradation [48, 49]. In addition, SUMO2/3-modified proteins were found to almost localize on nuclear bodies and cytoplasm [42]. According to these reports, we speculate that the interaction between p65 and SUMO2/3 may be a novel mechanism that regulates the stability of p65 via suppressing its degradation through ubiquitin-proteasome pathway in the cytoplasm.

A recent study showed that SUMO2/3 knockdown increased TNF-α-induced p65 transcriptional activity using a luciferase assay [21]. Kim EM also found that SUMO2 decreased p65 transcriptional activity [44]. Frauke Melchior proposed a model about the transcriptional regulation of SUMOylation, in which the SUMOylated transcriptional factors can recruit a inhibitory factor to its promoter to inhibit its downstream genes activation [50], such as CoREST1 and Sharp-1 [51, 52]. According to this model, SUMO proteins may mediate p65 SUMOylation and then recruit an inhibitor of p65 in nucleus. It was recently reported that SUMO2/3 inhibits nuclear import of p65 [44]. Additionally, we found that SUMO2 or SUMO3 increased the cytoplasmic p65. Consequentially, SUMO2/3 may suppress p65 transcriptional activity. SUMO system has been implicated in regulation of inflammation [53–56] and HCC [9–11, 57]. Our study further found that SUMO2/3 was involved in suppression of hepatoma cells proliferation.

## Conclusion

SUMO2/3-p65 interaction may be a novel mechanism involved in the transformation from chronic hepatitis B to HCC via stabilizing p65 in cytoplasm and limiting NF-κB activation, which might shed light on understanding the mechanisms of tumorigenesis and development.

## Consent

Written informed consent was obtained from the patient for the publication of this report and any accompanying images.

## Additional file

Additional file 1: **Expression of p65 in hepatoma cell lines.** p65 was detected by immunohistochemistry in HepG2 cells (A-B) and SMMC7721 cells (C-D) after treated with (B, D) or without (A, C) TNF-α (10 ng/ml, 30 min). (JPEG 1290 kb)

## Abbreviations

DAPI: 4′, 6-diamidino-2-phenylindole
HBV: Hepatitis B virus
HBx: Hepatitis B virus X protein
HCC: Hepatocellular carcinoma
SUMO: Small ubiquitin-related modifier
Ubc9: Ubiquitin-conjugating enzyme 9
