# Supplementary figures and images for: Small ubiquitin-related modifier 2/3 interacts with p65 and stabilizes it in the cytoplasm in HBV-associated hepatocellular carcinoma
Source: BMC Cancer. 2015 Oct 12;15:675. doi: 10.1186/s12885-015-1665-3 (PMC4603762; doi:10.1186/s12885-015-1665-3)

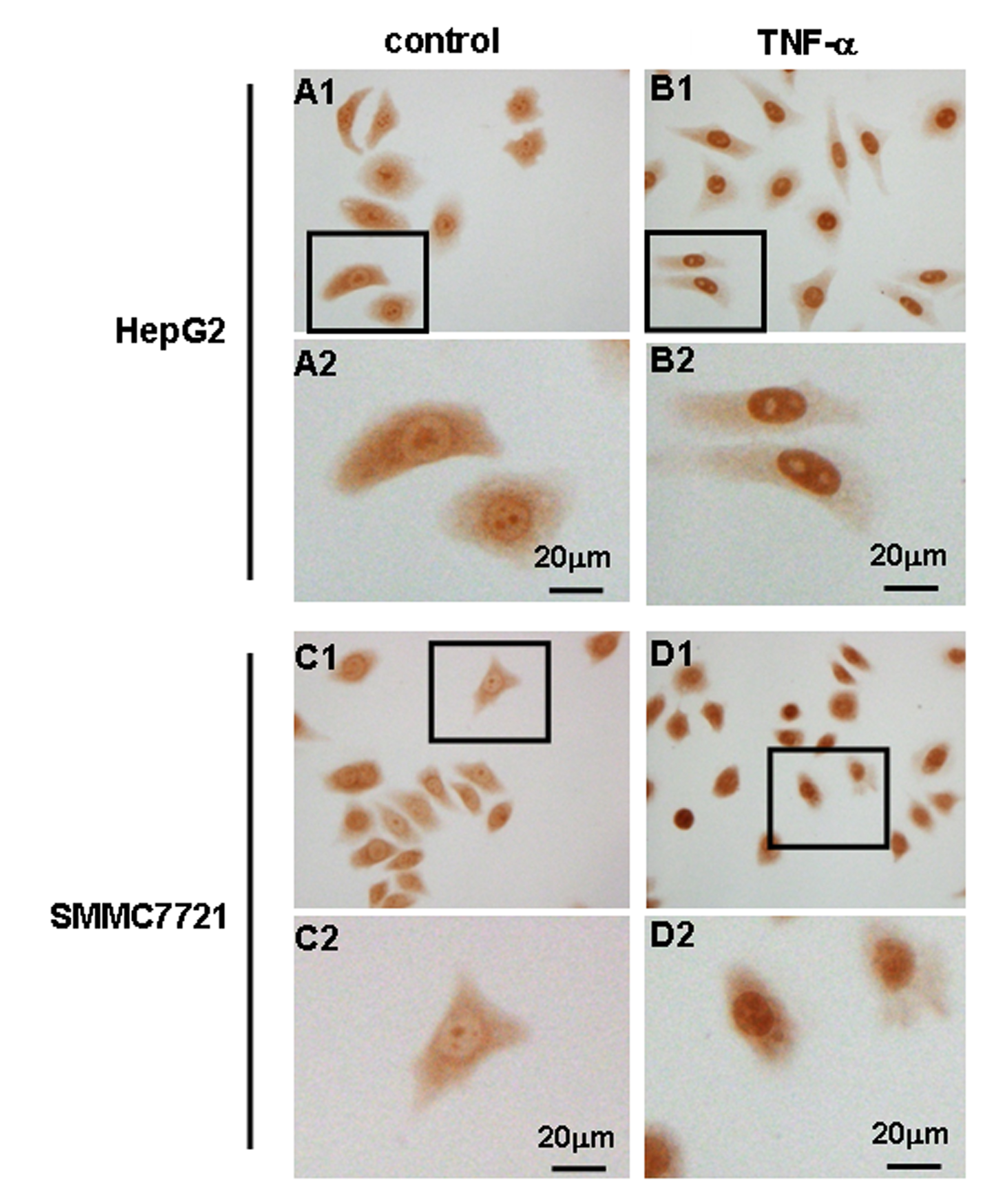

Supplement: Additional file 1: — Expression of p65 in hepatoma cell lines. p65 was detected by immunohistochemistry in HepG2 cells (A-B) and SMMC7721 cells (C-D) after treated with (B, D) or without (A, C) TNF-α (10 ng/ml, 30 min). (JPEG 1290 kb) [file 12885_2015_1665_MOESM1_ESM.jpeg]
